# A Strategy to Optimize the Mechanical Properties and Microstructure of Loess by Nano-Modified Soil Stabilizer

**DOI:** 10.3390/ma18194435

**Published:** 2025-09-23

**Authors:** Baofeng Lei, Xingchen Zhang, Henghui Fan, Shijian Wu, Changzhi Zhao, Wenbo Ni, Changhao Liu

**Affiliations:** 1 Northwest Engineering Corporation Limited, Power China, Xi’an 710100, China; 2 College of Water Resources and Architectural Engineering, Northwest A&F University, Yangling 712100, China2024055863@nwafu.edu.cn (C.L.)

**Keywords:** nano-stabilizer, stabilized soil, mechanical properties, microstructure

## Abstract

With the increasing demand for soil modification technologies in the field of civil engineering, this study employed cement-stabilized soil and MBER (Material Becoming Earth into Rock) stabilized soil as controls to investigate the modification effects of an N-MBER (nanosilica reinforced MBER) stabilizer on the mechanical properties and microstructure of loess. The mechanical and water stability characteristics of N-MBER-stabilized loess under varying moisture contents and compaction degrees were analyzed through unconfined compressive strength (UCS) tests, softening coefficient tests, falling-head permeability tests, and wet–dry cycle tests. Combined with scanning electron microscopy (SEM), X-ray diffraction (XRD), Fourier transform infrared spectroscopy (FT-IR), and nuclear magnetic resonance (NMR) techniques, the underlying mechanism of the N-MBER stabilizer in loess stabilization was thoroughly revealed. The results indicate that the N-MBER stabilizer significantly enhances the UCS and softening coefficient of loess. Particularly, under conditions of 28-day curing, a moisture content of 16%, and a compaction degree of 1, the compressive strength achieves a local optimum value of 3.68 MPa. Compared to soils stabilized with MBER stabilizers and cement stabilizers, the N-MBER-stabilized loess exhibits superior water resistance and microstructural density, with a significant reduction in the proportion of pore defects. Specifically, after five wet–dry cycles at a curing age of 28 days, the strength loss rates for MBER-stabilized soil and cement-stabilized soil were 24.4% and 27.54%, respectively, while that for N-MBER-stabilized soil was 18.23%, demonstrating its enhanced water resistance. Additionally, compared to cement-stabilized soil, the N-MBER-stabilized soil exhibited a 21.63% reduction in total pore number, with a 41.64% reduction specifically in large pores. The extremely small particle size and large specific surface area of the nanomaterial enable more effective interactions with soil particles, promoting hydration reactions. The resulting ettringite (AFt) and three-dimensional networked C-S-H gel tightly interweave with soil particles, forming a stable cemented structure. Compared to traditional concrete roads, stabilized soil roads enable the utilization of locally available materials and demonstrate a significant cost advantage. This study provides theoretical support and experimental evidence for the application of nanomaterials in loess improvement engineering.

## 1. Introduction

Approximately 6.6% of China’s total land area is characterized by typical loess landform regions. Due to their pronounced hydro-sensitive characteristics, this special geological material is prone to structural instability upon changes in moisture content, leading to a significant reduction in foundation bearing capacity and posing a potential threat to the stability of artificial structures [1,2,3]. Constrained by regional resource conditions, traditional concrete engineering faces dual limitations: scarcity of natural aggregate resources and high material transportation costs. Consequently, the engineering community widely adopts soil modification techniques using inorganic cementitious materials, incorporating cement-based or calcium-based stabilizers into foundation soils to enhance their mechanical properties [4,5]. However, given the unique mineral composition and pore structure characteristics of loess, conventional stabilization processes often encounter issues such as insufficient reactivity and low cementation efficiency [6].

To address this technical bottleneck, current engineering practice commonly employs multi-material composite modification strategies. By incorporating reinforcing components or activators, synergistic stabilization systems are constructed to enhance the strength development characteristics of modified soils, thereby meeting engineering design requirements [7,8]. In the field of novel soil stabilizer development, Fan’s research team [9] developed a multi-component cement-based composite stabilizer (MBER). A series of tests on loess improvement confirmed its effectiveness in enhancing soil engineering properties. In subsequent studies, the team [10,11] pioneered the extension of this technology to water-concentrating surface engineering. They conducted multi-dimensional research on the structural bearing capacity, hydraulic diversion efficiency, and construction standards of the stabilized soil matrix, establishing an integrated evaluation system encompassing material performance and construction control. Notably, Zhang et al. [12,13] optimized the MBER formulation by incorporating nano-silica-based materials. Microstructural test data indicated that this additive significantly shortens the strength development period of stabilized soil, providing an innovative solution for improving the early-stage mechanical properties of modified soil. The Kong team [14] systematically investigated the influence of nano-SiO_2_ dosage on the mechanical properties of reconstituted loess. However, quantitative analysis of the correlation between pore structure reorganization and cementation reaction pathways remains lacking. Thomas et al. [15] experimentally validated that nano-additives significantly enhance the compressive strength of cement-based soft clay composites. Nevertheless, the underlying microscopic mechanisms are not yet fully elucidated. Yong et al. [16] employed a rapid precipitation method to prepare magnesium hydroxide and calcium hydroxide nanoparticles for improving the engineering properties of tropical residual soils. The study revealed that after 14 days of curing, the UCS of soils treated with calcium hydroxide and magnesium hydroxide nanoparticles increased by 148.05% and 180.17%, respectively, compared to untreated soils, demonstrating their effectiveness as eco-friendly stabilizers. Kani et al. [17] systematically investigated the effects of binder type, nanosilica content, and curing age on the mechanical properties of stabilized soil. Their findings indicated that stabilized soil incorporating nanosilica exhibited enhanced UCS values, as nanosilica functions as a reactive powder that modifies the microstructure and promotes densification of binders. In recent years, the growing demand for soil modification technologies in civil engineering has led to increased acceptance of interdisciplinary applications of nanomaterials in soil improvement. This trend has stimulated market demand for nanomaterial products while potentially reducing engineering costs [18]. Although existing studies have enriched the research on performance enhancement and soil reinforcement using stabilizing agents, further exploration is still required regarding the key factors influencing nanomaterial-modified stabilizers in reconstructing soil properties, particularly in aggregate-scarce regions like the Loess Plateau. Moreover, previous research has primarily focused on soil consolidation through nano-modified cement/concrete or direct application of nanomaterials as primary stabilizers, there remains limited research on developing novel materials specifically aimed at enhancing the performance of soil stabilizers, such as nano-modified soil stabilizers. The mechanical properties and water stability of soils treated with novel nano-modified stabilizers warrant more comprehensive and systematic investigation.

This study focuses on the technological innovation aspects of nano-composite soil stabilizers. Employing an integrated research methodology combining laboratory experiments and multi-scale characterization techniques, we analyzed the mechanical properties and hydro-stability characteristics of nano-stabilized loess under varying moisture content and compaction conditions through unconfined compressive strength (UCS) tests, softening coefficient tests, falling-head permeability tests, and wet–dry cycle tests. Combined with scanning electron microscopy (SEM), X-ray diffraction (XRD), Fourier transform infrared spectroscopy (FT-IR), and nuclear magnetic resonance (NMR) techniques, we deeply revealed the mechanisms of nano-stabilizers in loess stabilization. This provides a theoretical basis for constructing an slope technology system in soil and water conservation engineering, while facilitating the engineering applications of nano-geomaterials in environmental geotechnical engineering.

## 2. Materials and Methods

### 2.1. Materials

**Figure 1:**
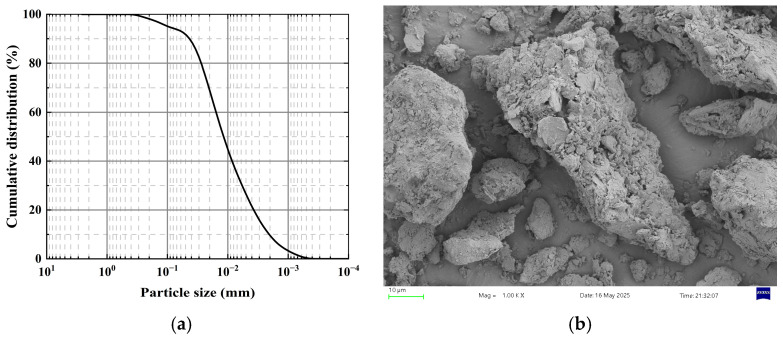
(**a**) Grain size distribution curve of the experimental soil; (**b**) Micro-morphology of the experimental soil.

**Figure 2:**
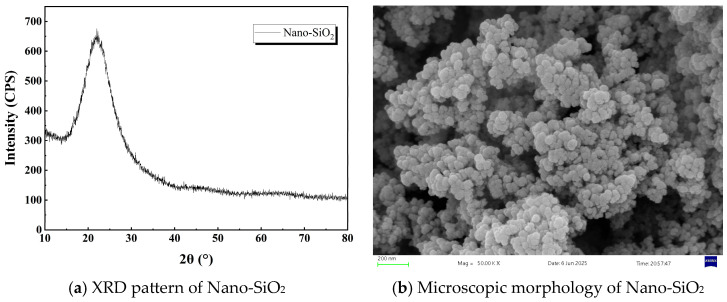
XRD patterns and microscopic morphology of Nano-SiO_2_.

The test soil was loess collected from Yan’an City in the hilly-gully region of China’s Loess Plateau, a typical collapsible soil widely distributed across this area. The particle size distribution curve and micromorphology of the test soil are shown in Figure 1, revealing sand content of 6.3%, silt content of 66.1%, and clay content of 27.6%, classifying it as low-plasticity clay. Basic physicochemical properties of the soil measured following the Standard for Geotechnical Testing Methods (GB/T 50123-2019) [19] are presented in Table 1. During the experiments, soil stabilized with the MBER (Material Becoming Earth into Rock) agent and soil stabilized with P.O 42.5 ordinary Portland cement served as the comparative groups. The MBER soil stabilizer, developed by Gao et al. [20], is a powdered, eco-friendly, inorganic cementitious material. The components of MBER curing agent and N-MBER (nanosilica reinforced MBER) curing agent are identical except for nano-SiO_2_, with the nano-SiO_2_ content in N-MBER curing agent being 2.5%. Core materials for the N-MBER stabilizer included P.O. 42.5 cement, sodium silicate, sodium sulfate, aluminum potassium sulfate, FDN water-reducing admixture, and nano-SiO_2_. Cement clinker was sourced from Qinlong Cement Factory in Xingping City, Shaanxi Province (China); sodium silicate was provided by the Guangdong Chemical Reagent Engineering Technology R&D Center (Guangdong, China); alum and sodium sulfate were supplied by Tianjin Zhiyuan Chemical Reagent Co., Ltd. (Tianjin, China); the water-reducing admixture came from Hefei Qiansheng Biotechnology Co., Ltd. (Anhui, China); nano-SiO_2_ was purchased from Shaanxi Dingyi Biotechnology Co., Ltd. (Shaanxi, China), confirmed as laboratory-grade high-purity, high-activity nanomaterial. The raw material proportions for the N-MBER stabilizer are listed in Table 2. The XRD patterns and micro-morphology of the nanosilica used in the tests are shown in Figure 2. The fundamental physical properties of the nanosilica were provided by the manufacturer, as detailed in Table 3 [21,22].

### 2.2. Test Methods

Based on previous studies [21,22], the testing program for unconfined compressive strength of N-MBER stabilizer-modified loess is presented in Table 4. The dosage of the N-MBER stabilizer was set at 12%.

The specimens were prepared according to the Test Procedures for Inorganic Binder Stabilized Materials in Highway Engineering (JTG E51-2009) [12]. Cylindrical specimens (Φ50 mm × 50 mm) were molded using the bidirectional static compaction method. The preparation procedure was as follows: undisturbed soil was crushed and passed through a 0.5 mm sieve, oven-dried, cooled to room temperature, then mixed with distilled water to achieve the target moisture content. The moistened soil was sealed in plastic bags and conditioned in a humidity chamber for 24 h to ensure uniform moisture distribution. Soil stabilizer was added to the moist soil and thoroughly mixed. The mixture was then compacted in custom-made molds to achieve specific compaction levels. Specimens were demolded using an ejector, with six parallel specimens prepared per group. All specimens underwent standard curing in a controlled chamber maintained at 20 ± 2 °C and >95% relative humidity until the designated curing ages.

To determine the optimal mix proportion and stabilization effectiveness of the nano-stabilizer, unconfined compressive strength (UCS) tests were conducted on nano-stabilized, conventional-stabilized, and cement-stabilized loess specimens using a WDW-100 microcomputer-controlled electro-hydraulic servo universal testing machine. The loading rate was set at 1 mm/min. To ensure data reliability, if the deviation between the extreme UCS value and the group average exceeded 15%, new specimens were prepared and tested until satisfactory consistency was achieved. Based on elastic mechanics theory [23], the compressive strength is calculated using Equation (1).

(1) $$\sigma =\frac{P_{max}}{A}$$

where *σ* represents the mean compressive strength (MPa), *P_max_* denotes the peak failure load (N), and *A* indicates the cross-sectional area of the specimen (mm^2^).

The softening coefficient test procedure followed established protocols [24]: (1) Cylindrical specimens (Φ50 mm × 50 mm) were prepared, with each test group containing 6 saturated and 6 dry specimens (12 total). (2) Specimens underwent standard curing (20 ± 2 °C, >95% humidity) until one day prior to testing. Saturation specimens were then immersed in 20 ± 2 °C water for 24 h with 2.5 cm water coverage. Dry specimens were oven-dried at 70 ± 3 °C for 24 h. (3) Unconfined compressive strengths of dry and saturated specimens were measured, with softening coefficient K_w_ calculated using Equation (2).

(2) $$K_{w}=\frac{f_{\mathrm{cu},1}}{f_{\mathrm{cu},2}}$$

where K_w_ denotes the softening coefficient; *f*_cu,1_ represents the unconfined compressive strength of stabilized soil in the saturated state (MPa); and *f*_cu,2_ corresponds to the unconfined compressive strength in the dry state (MPa).

Falling-head permeability tests were performed on specimens cured for 28 days to investigate the influence of compaction degree on the hydraulic characteristics of stabilized loess under different stabilizers. A conventional TST-55 permeameter was employed. Specimens were saturated in the sample chamber prior to testing. During each permeation cycle (with the water head dropping from 900 mL to 300 mL), the saturated hydraulic conductivity was calculated.

During the wet–dry cycling test, each cycle was set to 24 h, comprising a 16 h wetting phase and an 8 h drying phase. In the wetting phase, the specimens were fully immersed in water at 20 ± 2 °C to achieve saturation. In the drying phase, the specimens were placed in an oven at 45 °C.

**Figure 3:**
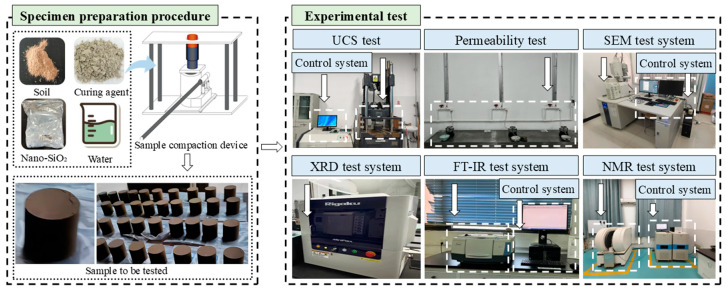
Specimen preparation procedures and testing methods.

Nuclear magnetic resonance (NMR) tests using a MacroMR12-110V-I analyzer (Suzhou Numai Analytical Instrument Co., Ltd., Jiangsu, China) were conducted on specimens subjected to 5 wet–dry cycles to examine the effects of stabilizer type and cyclic drying–wetting on pore distribution parameters. For microstructural analysis, fragments from UCS-tested specimens were placed in sealed containers filled with anhydrous ethanol to terminate hydration. Prior to testing, the fragments were dried in an oven at 60 °C until constant weight was achieved. Morphological and microstructural characteristics were analyzed by scanning electron microscopy (SEM) using a ZEISS field-emission microscope (Hitachi, Ltd., Tokyo, Japan). Phase composition and crystallinity were determined by X-ray diffraction (XRD) (Rigaku Miniflex600 benchtop diffractometer) (Tongce Technology Co., Ltd., Xi’an, China) with a 2θ range of 10–80° at a scan rate of 5°/min. Fourier transform infrared (FT-IR) spectroscopy (400–4000 cm^−1^) provided chemical bonding information based on characteristic absorption frequencies (Tongce Technology Co., Ltd., Xi’an, China). Figure 3 illustrates the specimen preparation and testing procedures [22].

## 3. Test Results and Analysis

### 3.1. Unconfined Compressive Strength Test Results

**Figure 4:**
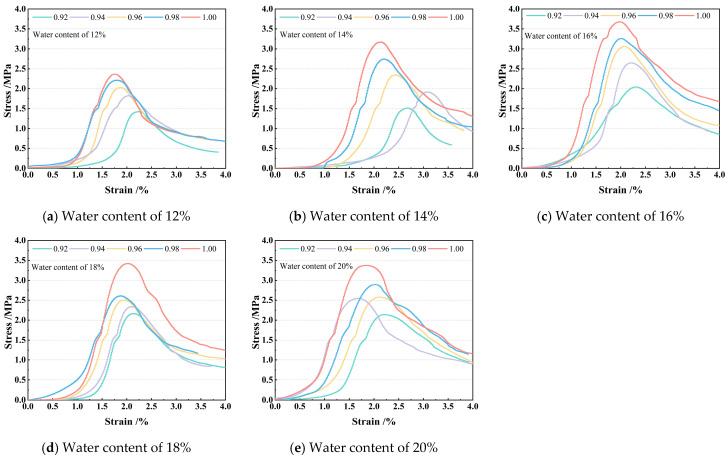
Axial load–displacement curve.

Unconfined compressive strength (UCS) tests were performed on specimens after curing. The stress–strain curves of the specimens are shown in Figure 4.

As shown in the stress–strain curves in Figure 4, at identical moisture contents, the peak strength of specimens increases with higher compaction degrees, and the curves shift toward the region of higher stress and smaller displacement. This indicates enhanced brittleness and increased load required to achieve equivalent displacement. Under identical compaction degrees, the peak strength initially increases then decreases with rising moisture content, reaching its maximum at 16% moisture content. These patterns primarily result from the physical effects of pore water and microstructural evolution. Near the optimal moisture content (16%), moderate water provides optimal capillary tension and bound-water cohesion, while its lubricating effect facilitates the formation of denser, more stable particle arrangements during compaction/loading, thereby maximizing bearing capacity and stiffness. At lower moisture content (12%), insufficient lubrication and cohesion lead to loose, fragile structures with reduced strength and susceptibility to brittle failure. At higher moisture content (20%), excess free water generates significant pore water pressure, substantially reducing the effective stress of the soil skeleton. Concurrently, the pronounced lubricating effect of water films diminishes interparticle friction and cohesion, resulting in soil softening, drastic strength reduction, and sustained plastic deformation. UCS test results are presented in Table 5.

**Figure 5:**
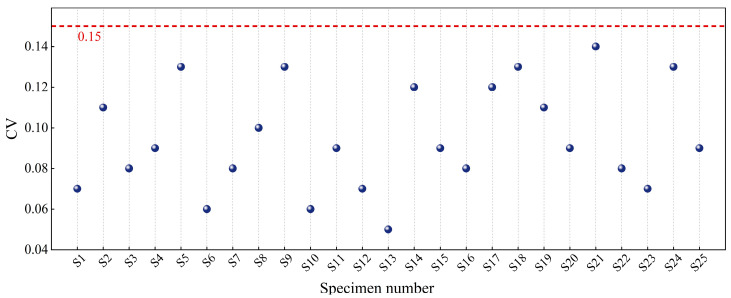
*CV* analysis for UCS measurements.

The coefficient of variation (*CV*) was used to assess the dispersion of mean UCS values across test conditions. Data with *CV* exceeding 15% were discarded as potentially unreliable. The *CV* calculation follows Equation (3).

(3) $$CV=\sqrt{\frac{\sum_{i=1}^{n}{\left(\sigma_{i}-\bar{\sigma }\right)}^{2}}{(n-1){\bar{\sigma }}^{2}}}$$

where $\sigma_{i}$ represents the measured UCS value of the *i*-th specimen (MPa), $\bar{\sigma }$ denotes the arithmetic mean of UCS (MPa), and *n* indicates the sample population. The computed *CV* values for UCS measurements, as illustrated in Figure 5, demonstrate statistically acceptable variability below 15% across all test groups, confirming data consistency.

**Figure 6:**
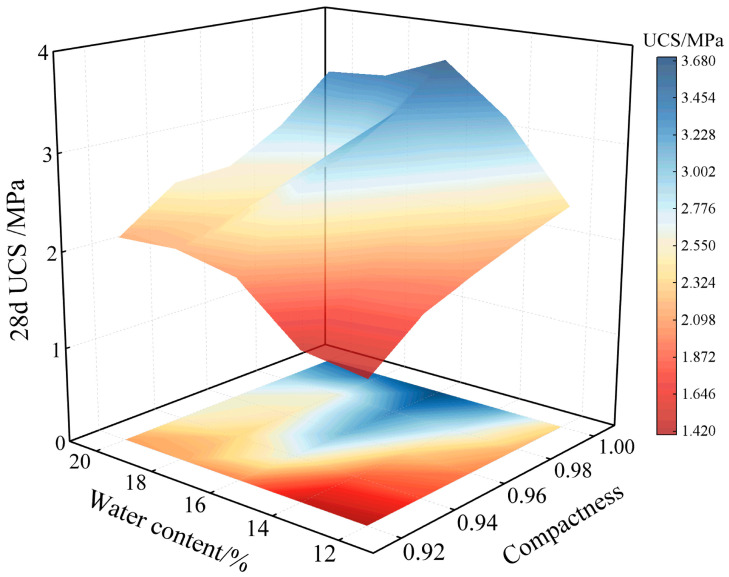
Relationship between different factors and 28 d compressive strength of nano-cured soil.

As indicated in Figure 5, the coefficients of variation (*CV*) for specimen UCS under all test conditions are consistently below 0.15, demonstrating low variability in the experimental results. Relationships between influencing factors and the 28-day compressive strength of nano-stabilized soil were established based on strength calculations in Table 5, as graphically presented in Figure 6.

As illustrated in Figure 6, the 28-day compressive strength of nano-stabilized soil increases progressively with higher compaction effort under constant moisture content. At 12% water content, specimens with a compaction degree of 0.92 exhibit a compressive strength of 1.42 MPa. When compaction degree increases to 1.0, the strength rises to 2.37 MPa, representing a 66.9% enhancement. This phenomenon is attributed to progressively reduced soil porosity and enhanced particle-to-particle contact at elevated compaction levels, which expands the contact area for curing agents and amplifies the effects of cement hydration products and nano-SiO_2_. Previous studies [22] have demonstrated that nano-SiO_2_ possesses exceptionally high specific surface area, effectively filling interparticle voids to improve overall density while mitigating the negative impact of voids on mechanical properties, thereby significantly enhancing compressive strength.

Under constant compaction degree, the compressive strength of nano-stabilized soil demonstrates an initial increase followed by a decrease with rising water content. At a compaction degree of 1.0 and water content of 16%, specimens attain a peak compressive strength of 3.68 MPa. Previous studies [25] indicate water acts as both a lubricant and reaction medium in soil, influencing pore structure, interparticle bonding, and hydration reactions of curing agents. When water content reaches the optimal range (16%), enhanced particle lubrication promotes complete hydration of cement and nano-silica. Cement hydration products effectively bond with soil particles, filling interparticle voids to form a denser microstructure, thereby maximizing compressive strength.

In summary, nano-stabilized soil achieves peak performance at 16% water content and 1.0 compaction degree. To compare its efficacy with conventional curing agents and P.O. 425 ordinary Portland cement-stabilized soil, subsequent tests were designed under these optimized conditions as detailed in Table 6.

**Figure 7:**
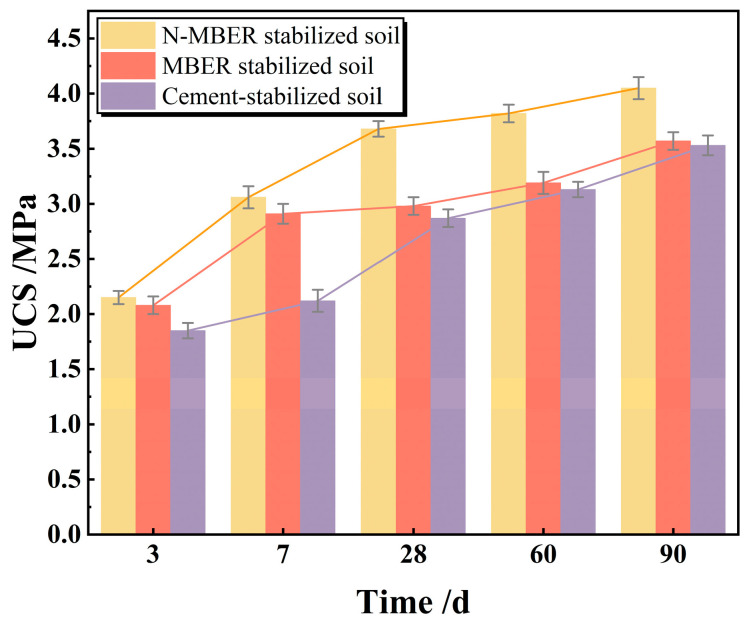
Relationship between age of maintenance and compressive strength of cured soils.

Upon completion of the curing period, UCS tests were performed on the specimens, and the results are illustrated in Figure 7.

As illustrated in Figure 7, specimens stabilized with different curing agents exhibit progressively increasing compressive strength with extended curing age, demonstrating pronounced enhancement during initial curing stages followed by gradually stabilizing growth. For N-MBER stabilized loess, compressive strength rises significantly from 2.15 MPa to 3.68 MPa within the first 28 days, primarily due to progressive chemical reactions and physical adsorption that form stable hydration products. Subsequently, strength increases marginally from 3.68 MPa to 4.05 MPa between 28 and 90 days, indicating approaching reaction saturation and stabilized strength development. When MBER stabilizers are employed, compressive strength shows rapid growth from 2.08 MPa to 2.91 MPa (+0.83 MPa) during 0–7 days, reflecting effective early-stage hydration. From 7 to 28 days, strength increases minimally by 0.07 MPa (2.91→2.98 MPa), suggesting near-completion of primary reactions. Although strength further rises to 3.57 MPa (+0.59 MPa) between 28 and 90 days, the decelerating growth rate confirms curing nearing saturation with diminishing returns on extended curing. For cement-stabilized loess, early strength increases from 1.85 MPa to 2.12 MPa (+14.6%) during 0–7 days through hydration of C_3_S and C_2_S forming initial C-S-H gels that fill soil voids. Substantial growth to 2.87 MPa (+35.4%) occurs at 7–28 days as continued C-S-H formation enhances particle cementation. Final strength reaches 3.53 MPa between 28 and 90 days with markedly reduced growth, indicating near-complete hydration of reactive cement components.

### 3.2. Softening Coefficient Test Results and Analysis

The softening coefficient, defined as the ratio of saturated compressive strength to dry compressive strength of stabilized soil, quantifies material water resistance by reflecting strength reduction after water immersion. Section 3.1 results indicated minimal unconfined compressive strength development in stabilized loess after 28 d of curing. Consequently, a 28 d curing period was adopted for subsequent tests.

The experimental results of the softening coefficient tests for soils stabilized with different curing agents are summarized in Table 7.

As indicated in Table 7, stabilized loess exhibits significant strength reduction after water immersion compared to its dry state. The softening coefficients were measured as 0.339 (66.1% strength reduction) for N-MBER stabilized loess, 0.296 (70.4% reduction) for MBER stabilizer-treated loess, and 0.286 (71.4% reduction) for cement-stabilized loess. This strength degradation primarily results from partial structural disintegration upon water exposure. Notably, N-MBER stabilized loess demonstrated superior softening resistance compared to MBER and cement-based stabilizers. Previous studies reveal that MBER and cement-based stabilizers form relatively loose cementation structures through mineral reactions with loess, leaving substantial residual pores [21,26]. These interconnected pore networks facilitate water infiltration under saturated conditions, compromising overall water resistance. In contrast, nano-stabilizers feature smaller particle sizes and higher specific surface areas, enabling effective pore-filling at microscale. This mechanism enhances soil compactness and substantially inhibits water penetration potential [27]. Concurrently, N-MBER stabilizers establish stronger particle cementation, transforming the originally loose loess matrix into a stabilized microstructure with enhanced water-blocking capacity. This structural refinement significantly improves hydraulic stability through optimized pore connectivity reduction.

### 3.3. Falling-Head Permeability Test Results and Analysis

**Figure 8:**
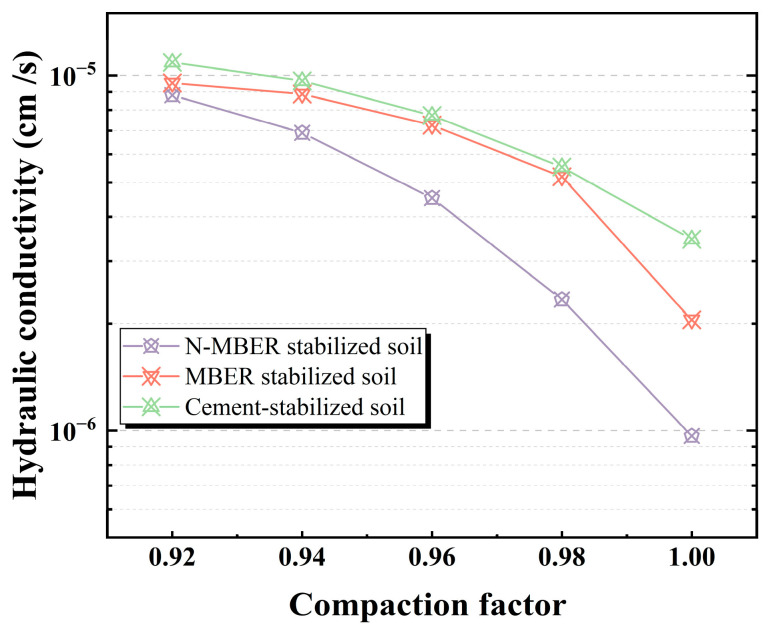
Relationship between permeability coefficient and compaction degree of stabilized soils.

Compaction degree governs the saturated permeability coefficient of compacted loess. As shown in Figure 8, identical molding water content specimens exhibit greater saturated permeability coefficients at lower compaction degrees. Reduced compaction promotes looser aggregate arrangements, enlarges interparticle pore volumes, and enhances connectivity of seepage pathways, consequently increasing permeability [28]. Conversely, higher compaction degrees induce tighter inter-aggregate contacts, diminish pore dimensions, and impair seepage path connectivity, thereby reducing permeability.

Under identical compaction conditions, the efficacy of three stabilizers is as follows: nano-stabilizer > conventional stabilizer > cement stabilizer. The nano-stabilizer demonstrates superior impermeability, particularly at high compaction degrees (>0.96), where its permeability coefficient significantly undercuts its counterparts, highlighting exceptional seepage prevention potential. This performance stems from nano-SiO_2_ particles leveraging their nanoscale size and substantial specific surface area to penetrate and fill minute pores/microcracks. Through physicochemical interactions (e.g., adsorption, reactions), they establish a dense micro-barrier layer, achieving minimal permeability at equivalent compaction. These pore-sealing advantages intensify at higher compaction degrees.

### 3.4. Wetting–Drying Cycle Test Results and Analysis

**Figure 9:**
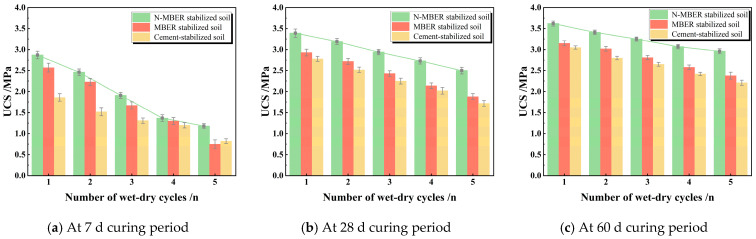
Effect of dry–wet cycles on the peak strength of stabilized soil.

Figure 9 reflects the variation in peak strength of specimens subjected to wetting–drying cycles at different curing ages. Analysis reveals that strength progressively declines with increasing cycles, exhibiting marked reduction within the first three cycles. This deterioration stems from synergistic physical disruption and chemical dissolution during moisture migration. During drying phases, evaporation induces intense capillary tension that pulls soil particles apart, generating shrinkage stresses that initiate microcracks at weak points. Concurrently, soluble salts migrate to the surface and crystallize, exerting expansive pressure on pore walls that propagates microcracks.

In wetting phases, water re-infiltrates the soil, causing clay minerals (e.g., montmorillonite) to swell and expand existing microcracks. Critically, water dissolves cementitious components—particularly portlandite (Ca(OH)_2_) in cement-stabilized soils—compromising bonding network integrity [29]. After multiple cycles, microcracks coalesce into macrocracks (reducing load-bearing sections), cementitious loss weakens interparticle bonds, and porosity markedly increases. These three degradation mechanisms collectively drive systematic strength reduction [30].

Comparative analysis demonstrates N-MBER-stabilized soil’s superior durability. At 28-day curing, after five cycles, MBER- and cement-stabilized soils exhibit strength losses of 24.4% and 27.54%, respectively, while N-MBER-stabilized soil shows only 18.23% loss. This enhanced water resistance originates from nano-SiO_2_ particles (1–100 nm) penetrating sub-micron pores inaccessible to MBER stabilizers. These ultrafine particles form densely packed physical barriers that disrupt capillary water pathways through pore-filling and microcrack-sealing mechanisms. By fundamentally inhibiting moisture migration, nano-stabilized soils maintain denser structures throughout cycling [31,32].

## 4. Microstructural Characteristics and Stabilization Mechanisms

### 4.1. XRD Results and Analysis

**Figure 10:**
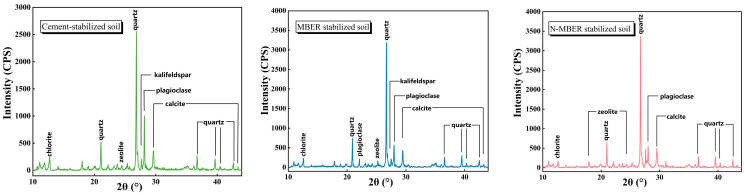
XRD patterns of specimens treated with different stabilizers.

To investigate the influence mechanism of nano-SiO_2_ on the mineral composition of stabilized soil, comparative X-ray diffraction (XRD) analyses were conducted on N-MBER-stabilized soil, MBER-stabilized soil, and cement-stabilized soil after 28 days of curing. These specimens were prepared at 16% water content and had a relative compaction of 1. The resulting mineral phase characteristics are presented in Figure 10 [22]. The XRD patterns of all three soils exhibited diffraction peaks of quartz, kaolinite, plagioclase, and calcite. Quartz showed the highest peak intensity, indicating its strongest diffraction capability. However, the quartz peak in cement-stabilized soil was substantially lower than in N-MBER and MBER stabilized soils. This reduction may arise from (i) post-drying sample heterogeneity affecting quartz content, or (ii) overlapping peaks with other phases diminished during hydration—such as reactive SiO_2_ consumed to form calcium silicate hydrate (C-S-H) gel [33]. No significant differences emerged in mineral types among the three soils, suggesting nano-SiO_2_ minimally alters mineral composition. Instead, it primarily accelerates hydration/hydrolysis reactions via size effects and interface activation of inert minerals, thereby modifying macroscopic physico-mechanical properties [21]. Notably, C-S-H gel (amorphous phase) lacks detectable XRD peaks, while cement contains dicalcium silicate (C_2_S) and tricalcium silicate (C_3_S) that readily form C-S-H. Thus, the broad hump between 30 and 40° may correspond to this phase [34]. The hydration product peak intensities in N-MBER- and MBER-stabilized soils slightly exceeded those in cement-stabilized soil, indicating nano-SiO_2_ enhances reaction kinetics to generate more hydration products at equivalent curing ages.

### 4.2. FT-IR Results and Analysis

**Figure 11:**
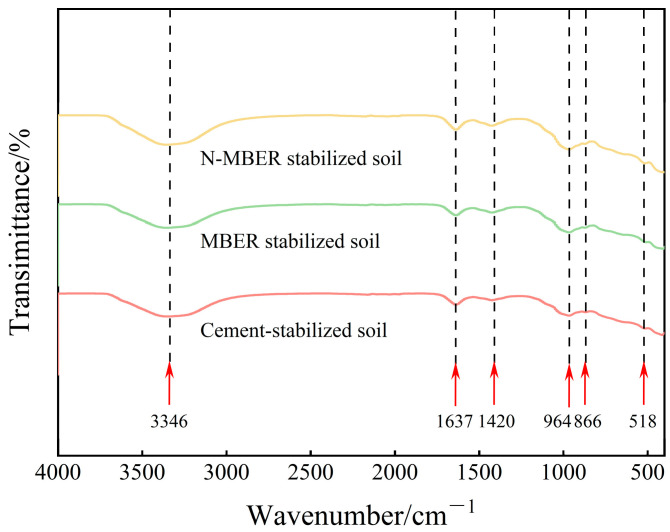
FT-IR spectra of specimens treated with different stabilizers.

FT-IR analysis provides information on the chemical bonds in the samples through the characteristic absorption frequencies of infrared light by different chemical bonds. The FT-IR spectra of the three types of stabilized soil (prepared at 16% water content and relative compaction of 1) after 28 days of curing are displayed in Figure 11. Analysis revealed a broad absorption peak near 3346 cm^−1^ in all specimens, corresponding to O-H stretching vibrations in Si-OH groups, indicating adsorbed water molecules on sample surfaces. An absorption peak observed near 1637 cm^−1^ represents H-O-H bending vibrations [34]. These two peaks signify weakly bound water typically present during hydration reactions, potentially occurring during the formation of hydration products such as C-A-S-H gel. Absorption peaks near 866 cm^−1^ and 964 cm^−1^ are attributed to Si-O-Si and Si-O-Al stretching vibrations, with this spectral region (720–1250 cm^−1^) primarily indicating strength-contributing phases including C-S-H and C-A-S-H gels [35]. As reported in [36], peak width inversely correlates with gel crystallinity (sharper peaks denote higher crystallinity). Comparative analysis of the three soils shows N-MBER-stabilized soil exhibits the sharpest peaks in this region, indicating the highest hydration reaction extent and most abundant cementation product formation. The characteristic peak at 518 cm^−1^ arises from Si-O bending vibrations, representing a spectral signature of SiO_2_. An absorption peak near 1420 cm^−1^ corresponds to C-O bending and asymmetric stretching vibrations, possibly due to CO_2_ absorption by the stabilizer during specimen preparation leading to calcium carbonate formation. Notably, N-MBER-stabilized soil also demonstrates the sharpest peak at this location.

### 4.3. SEM Results and Analysis

Following UCS testing, specimens undergo SEM cross-sectional observation and EDS energy dispersive spectroscopy analysis to examine surface microstructure while simultaneously analyzing chemical element distribution.

**Figure 12:**
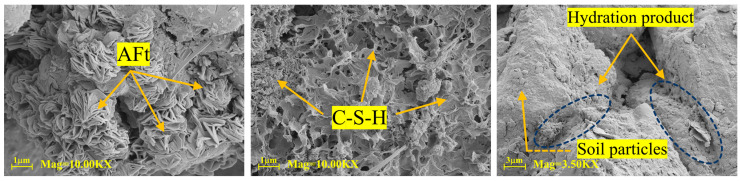
Microstructure of N-MBER-stabilized specimen.

**Figure 13:**
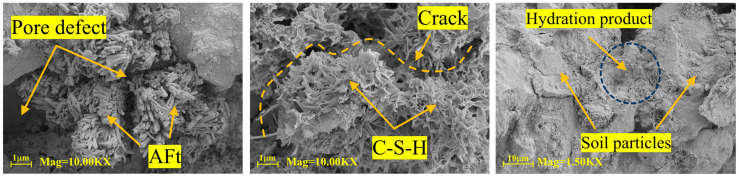
Microstructure of MBER stabilizer-treated specimen.

**Figure 14:**
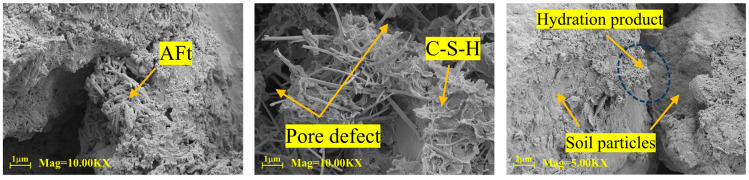
Microstructure of cement-stabilized specimen.

Following 28 days of curing, the morphology of microscopic products in loess specimens stabilized with different agents (prepared at 16% water content and relative compaction of 1) is displayed in Figure 12, Figure 13 and Figure 14. As shown in Figure 12, at 28 days of curing, SEM analysis of N-MBER-stabilized loess reveals abundant hydration products formed between soil particles, including rod-shaped ettringite (AFt) and three-dimensional C-S-H networks. These hydration products interweave with soil particles spatially, indicating that the N-MBER stabilizer enhances interparticle cementation at the microscale, forming a compact structure. The ultra-fine particle size and relatively large specific surface area of nanomaterials promote stronger interactions with soil particles, accelerating hydration reactions. By improving reaction activity and providing additional bonding sites, the N-MBER stabilizer generates denser and more homogeneous hydration products, thereby enhancing the strength and stability of loess. In contrast, Figure 13 demonstrates that MBER stabilizer produces clustered ettringite and flaky C-S-H between soil particles, with lower quantities of hydration products and weaker cementation compared to N-MBER-stabilized specimens. This discrepancy arises from the larger particle size and lower reactivity of ordinary stabilizers, resulting in less abundant and uniformly distributed hydration products. The loosely clustered structures provide insufficient binding force for effective soil particle integration. Conversely, Figure 14 reveals that cement-stabilized loess generates both C-S-H and ettringite, but exhibits inferior compactness and distribution of hydration products at the microstructural level due to weaker inter-product interactions. The scarcity of hydration products between soil particles leads to lower cementation strength, consequently diminishing overall strength and stability relative to N-MBER-stabilized loess.

Therefore, based on Figure 12, Figure 13 and Figure 14, the N-MBER stabilizer reacts with soil particles at a finer scale, producing more abundant hydration products. Through its uniform and compact microstructure, it effectively binds soil particles, significantly enhancing soil strength. While MBER and cement stabilizers can stabilize loess, their effectiveness in improving cementation strength and stability remains inferior to the N-MBER stabilizer.

**Figure 15:**
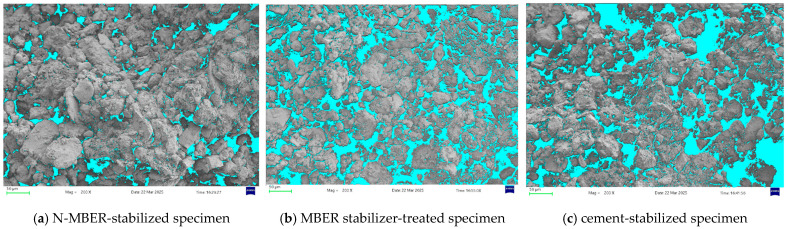
Cross-sectional microstructural morphology of stabilized specimens (200× Mag).

**Figure 16:**
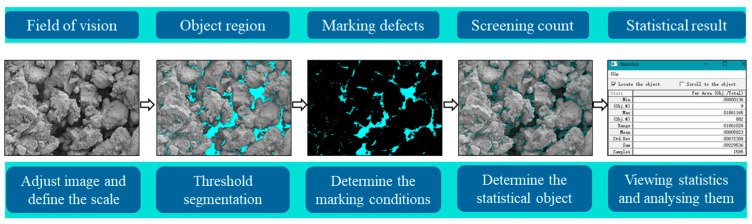
Statistical process of void defect area.

Figure 15 presents SEM images of three stabilized soil specimens after a 28-day curing period. Observation reveals that the N-MBER-stabilized soil exhibits the densest microstructure, where hydration products not only fill internal pores between soil particles but also form enveloping layers on particle surfaces, enhancing interparticle bonding and resulting in a more compact overall structure. In contrast, the cement-stabilized soil displays a relatively loose microstructure with numerous unfilled fine pores and fissures surrounding soil particles. For quantitative analysis of the pore proportion in the three types of stabilized soils, Image-Pro Plus software (Version 6.0) was employed to calculate the pore area in SEM images captured at the same magnification. The calculation formula for pore defects is given in Equation (4), with the computational procedure illustrated in Figure 16. The calculated average pore defect area ratios are presented in Table 8, where the final results represent the mean values obtained from three SEM micrographs.

(4) $$R_{vd}=\frac{A_{vd}}{A}$$

where $A_{vd}$ represents the void defect area within the FOV (μm^2^), and A corresponds to the total FOV area (μm^2^).

Combining the results from Figure 15 and Table 8, the N-MBER-stabilized soil specimen exhibits a denser microstructure, with its void defect area ratio being only one-fifth of that in cement-stabilized specimens within identical fields of view. This discrepancy arises from the larger particle size of cement, which limits its pore-filling effectiveness, resulting in higher porosity and coarser hydration products. According to prior studies [37,38], nano-SiO_2_ (particle size: 15–30 nm) possesses a specific surface area of 300–400 m^2^/g—50 times greater than ordinary cement. Consequently, the N-MBER stabilizer not only fills pores but also accelerates hydration reactions to produce additional gels, while its nanoparticles physically clog finer pores, thereby enhancing structural compactness.

**Figure 17:**
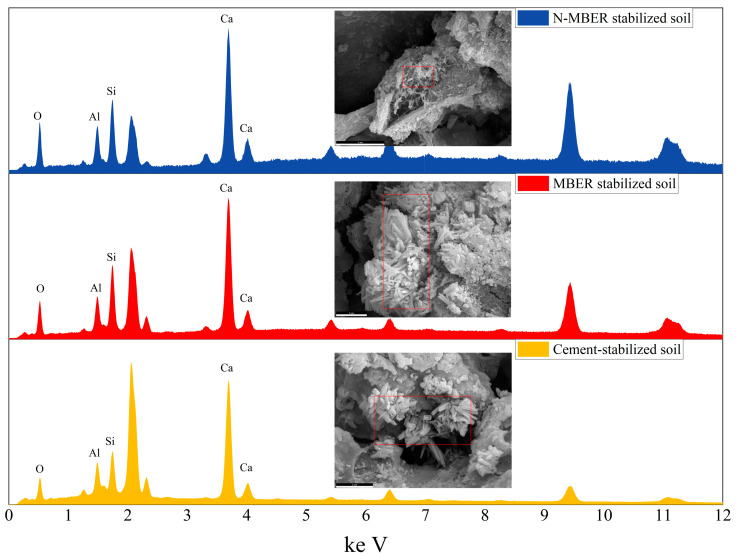
EDS test results of loess specimens stabilized with different stabilizers (The red square represents the location of the test).

Microstructural examination and EDS analysis were performed on fracture surfaces of the three stabilized soils. By analyzing phase-boundary structures and crystalline composition variations, this study investigates the interface reconstruction process and mechanism of N-MBER stabilizers in soil units, with analytical results presented in Figure 17.

As illustrated in Figure 17, a randomly selected point on the stabilized soil specimen (magnified 10,000×) exhibits primary elements including O, Si, Al, and Ca. The elemental composition confirms hydration products comprising ettringite (AFt), calcium silicate hydrate (C-S-H) gel, and calcium aluminosilicate hydrate (C-A-S-H) gel. These reaction products provide cohesive forces between soil particles, imparting initial strength to the material. Given that cement constitutes the primary component across all three stabilizers, its calcareous and siliceous constituents react with minerals in loess during stabilization. The resulting AFt, C-S-H, and C-A-S-H form dense crystalline structures on particle surfaces, creating robust cementitious layers. These hydration products not only enhance interparticle bonding but also fill micropores between soil particles, thereby improving soil compactness and strength [39,40,41].

### 4.4. NMR Results and Analysis

**Figure 18:**
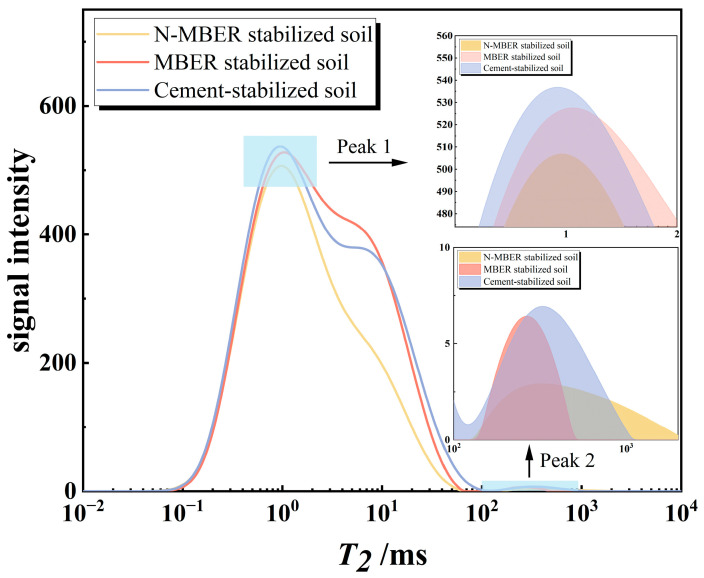
*T*_2_ distribution curves of the three stabilized soils.

NMR curves reflect pore distribution characteristics within soil. Figure 18 presents T_2_ spectral distributions of three stabilized soils after five wet–dry cycles. Based on T_2_ spectral theory, relaxation times are categorized as follows: 0.1–1 ms (micro-fissures), 1–100 ms (medium fissures), and 100–1000 ms (macro-fissures), with pore characteristic parameters detailed in Table 9. Figure 18 demonstrates significantly fewer pores across all size ranges in N-MBER-stabilized soil compared to cement-stabilized soil, with total porosity reduced by 21.63% and macro-pores decreased by 41.64%. This indicates that nano-SiO_2_ fills internal pore structures via micro-aggregate effects, enhancing overall compactness. Additionally, the initially higher density of N-MBER-stabilized soil mitigates mechanical degradation during wet–dry cycles. Compared to MBER-stabilized soil, N-MBER-stabilized soil exhibits similar macro-pore quantities but significantly fewer micro- and medium-pores.

## 5. Cost

The cost competitiveness of nano-stabilizer materials in constructing soil and water conservation infrastructure, compared with traditional building materials, serves as a crucial indicator for their practical application and widespread adoption. Taking a laterite soil road project as an example (road dimensions: 50 m × 3.5 m × 0.2 m; compacted density of stabilized soil: 1.8 g/cm^3^) [21,24], the engineering cost calculation is presented in Table 10. The required stabilized soil amounts to approximately 63 tons, while the total mixture is calculated as 100 tons for comparison, consisting of 15% nano-stabilizer (15 tons) and 85% laterite soil (85 tons). According to Table 10, the material cost of stabilized soil shows significant savings of about CNY¥17,800 compared with concrete, representing a cost reduction exceeding 70%. This approach enables the utilization of local materials, effectively lowering project costs.

## 6. Conclusions

This study systematically analyzes the mechanical properties and microstructure of nano-stabilizer modified loess, yielding the following conclusions.

(1) The compressive strength of N-MBER-stabilized soil initially increases then decreases with rising moisture content, while gradually increasing with higher compaction. At 28 days of curing under 16% moisture content and compaction degree of 1, the compressive strength reaches a local optimum of 3.68 MPa.
(2) Extended curing time enhances soil strength, with growth rate decelerating beyond 28 days. Compared to MBER and cement-stabilized soils, N-MBER-stabilized loess demonstrates superior water resistance and microstructural density, exhibiting significantly reduced void defect ratios. After five wet–dry cycles at 28-day curing, strength loss rates reach 24.4% for MBER and 27.54% for cement-stabilized soils, versus 18.23% for N-MBER-stabilized soil. Additionally, N-MBER-stabilized soil shows 21.63% fewer total pores and 41.64% fewer macro-pores than cement-stabilized counterparts.
(3) The nanomaterial’s ultra-fine particle size and high specific surface area facilitate enhanced interactions with soil particles, accelerating hydration reactions. Generated ettringite (AFt) and three-dimensional networked C-S-H gels intertwine tightly with soil particles, forming dense stabilization layers that reduce void defects and establish a stable cemented framework, thereby enhancing overall stability.
(4) Compared to traditional concrete roads, nano-stabilized soil roads can utilize local soil as raw material, offering a significant cost advantage over traditional building materials.

## Figures and Tables

**Table 1:** Physical and chemical properties of Yan’an loess.

Properties	Test Result
Classification	CL
Specific gravity, *G_S_*	2.68
Maximum dry density, *ρ*_max_ (g/cm^3^)	1.76
Optimum moisture content, *w*_opt_ (%)	16.8
Liquid limit, *W_L_* (%)	31.3
Plastic limit, *W_P_* (%)	18.2
Grain-size distribution	
Clay content (%)	27.6
Silt content (%)	66.1
Sand content (%)	6.3

**Table 2:** N-MBER stabilizer ingredients and SO_3_ content in the test.

Ingredients	Cement Clinker	Fly Ash	Gypsum	Active Agent	Nano-SiO_2_	N-MBER Stabilizer
Content %	82.5	11	3	1	2.5	100
SO_3_ content	2.01	0.26	1.40	0	0	3.67

**Table 3:** Physical properties of nano-silica.

Purity (%)	APS (nm)	SSA (m^2^/g)	Morphology	Bulk Density (g/cm^3^)	True Density (g/cm^3^)
99.9	30	600	spherical	0.08	2.2~2.6

**Table 4:** Testing program.

Factors	Levels
Water content/%	12, 14, 16, 18, 20
Degree of compaction	0.92, 0.94, 0.96, 0.98, 1.0
Curing period/d	28

**Table 5:** Unconfined compressive strength.

Specimen Number	Water Content/%	Degree of Compaction	28 d UCS /MPa	Specimen Number	Water Content/%	Degree of Compaction	28 d UCS /MPa
S1	12	0.92	1.42	S14	16	0.98	3.26
S2	12	0.94	1.83	S15	16	1.00	3.68
S3	12	0.96	2.03	S16	18	0.92	2.17
S4	12	0.98	2.21	S17	18	0.94	2.34
S5	12	1.00	2.37	S18	18	0.96	2.51
S6	14	0.92	1.51	S19	18	0.98	2.61
S7	14	0.94	1.92	S20	18	1.00	3.42
S8	14	0.96	2.34	S21	20	0.92	2.14
S9	14	0.98	2.75	S22	20	0.94	2.55
S10	14	1.00	3.17	S23	20	0.96	2.58
S11	16	0.92	2.04	S24	20	0.98	2.89
S12	16	0.94	2.65	S25	20	1.00	3.37
S13	16	0.96	3.06				

**Table 6:** Test program for cured soils with different curing agents.

Type of Stabilizer	Stabilizer Content/%	Moisture Content/%	Compaction Degree	Curing Age/d
N-MBER stabilizer	12%	16%	1	3, 7, 28, 60, 90
MBER stabilizer				
Cement-based stabilizer				

**Table 7:** Softening coefficient test results of soils stabilized with different curing agents.

Type of Stabilizer	Softened Strength/MPa	Dry Strength/MPa	Softening Coefficient
N-MBER stabilizer	3.60	10.62	0.339
MBER stabilizer	2.77	9.37	0.296
Cement-based stabilizer	2.53	8.86	0.286

**Table 8:** The results of the proportion of pore area in the SEM field of view.

Type of Stabilizer	Average Pore Defect Area/μm^2^	Average Proportion of Pore Defect Area/%
N-MBER stabilizer	21,295.23	8.86
MBER stabilizer	59,546.19	24.05
Cement-based stabilizer	100,594.71	40.62

**Table 9:** Pore size distribution characteristics of the three stabilized soils, with *CV* (Coefficient of Variation, calculated as the ratio of the standard deviation to the mean) values.

Stabilizer Type	Total Pores	***CV***	Micropores	***CV***	Medium-Pores	***CV***	Macro-Pores	***CV***
N-MBER stabilizer	21,859.548	0.058	19,864.154	0.059	1919.204	0.069	76.190	0.096
MBER stabilizer	27,468.048	0.075	23,677.536	0.078	3714.286	0.062	76.226	0.072
Cement-based stabilizer	27,892.730	0.060	23,358.953	0.056	4403.230	0.083	130.547	0.066

**Table 10:** Construction cost of stabilized soil road engineering.

Project	Unit Price/RMB	Cured Soil Road		Concrete Road	
		Quantity/ton	Price/RMB	Quantity/ton	Price/RMB
Nano-curing agent	480	15	7200	0	0
Red soil	0	85	0	0	0
Aggregate	250	0	0	100	25,000

## Data Availability

The original contributions presented in this study are included in the article. Further inquiries can be directed to the corresponding authors.
